# Management and current status of spinal muscular atrophy: a retrospective multicentre claims database analysis

**DOI:** 10.1186/s13023-019-1287-y

**Published:** 2020-01-10

**Authors:** Josep Darbà

**Affiliations:** Department of Economics, Universitat de Barcelona, Diagonal 696, 08034 Barcelona, Spain

**Keywords:** Spinal muscular atrophy, Disease management, Patient demographics, Claims database analysis, Spain

## Abstract

**Introduction:**

The interest in patient demographics and disease management has increased in the past years due to their utility in developing measures that allow healthcare providers to reflect disease complexity.

**Objective:**

To revise the current status of spinal muscular atrophy in the region of Catalonia, and to validate the utility of the database for this aim.

**Methods:**

Five hundred twenty-four patients diagnosed with a spinal muscular atrophy were identified in the region of Catalonia via the novel program of data analysis for research and innovation in health (PADRIS). Patient records included in the analysis corresponded to primary care, hospital, emergency room, extended care and mental health admissions between 2007 and 2017.

**Results:**

58.02% of patients with a SMA diagnosis were males while 40.84% were females. Average age of diagnosis was 38.31 ± 24.49 years ±SD. Significantly lower was the age of diagnosis of spinal muscular atrophy type I, 1.81 ± 3.01 years. An average of 22 patients died per year during the study period, with a mean decease age of 62.96 ± 25.41 years. Patients were generally attended in hospitals, and the use of healthcare resources was focused on resolving respiratory issues and scoliosis. The highest ratio of admissions per patient was registered in those aged 0 to 4 years. Patients presented a higher risk than the general population and a higher frequency of multimorbidites.

**Conclusions:**

Patients exhibited similar characteristics to prior European studies. Multiple admissions in younger patients, mostly due to respiratory issues, have a central role in increasing medical costs of SMA. Equally, the higher risk of patients and increased number of multimorbidity groups translate in an elevated number of admissions in health centres and ER, deriving in higher expenses.

## Introduction

Spinal muscular atrophy (SMA) describes a group of hereditary and recessive neuromuscular disorders caused by the degeneration of anterior horn cells resulting in muscle atrophy and weakness [1]. Contrarily, progressive SMA refers to a non-hereditary condition [2]. Distinct SMA types are characterized by different degrees of motor function and age of onset. SMA-I or Werdnig-Hoffmann syndrome, is diagnosed during the first 6 months of life, and causes severe development limitations. SMA-II has its onset between 7 and 18 months of age, before the child is able to walk. SMA-III or Kugelberg-Welander syndrome is divided in two subtypes; type IIIa is diagnosed in children of 2 to 3 years of age, who will have orthopaedic issues; and type IIIb appears between 3 years of age and late adolescence. These patients have a normal motor development [3, 4]. SMA-IV and zero have been described a posteriori and refer to SMA with an adult onset and the disease affecting children in the first weeks of life respectively [3, 5].

Considering all disease manifestations, the paediatric population remains the most affected by this disorder. Additionally, SMA-0 and SMA-I present the most severe forms of the condition, which determines the importance of paediatric care [5]. SMA is estimated to affect around 1 in 10,000 live births [6], with irregularities among regions and ethnic groups [7], thus the importance to evaluate disease demographics at a regional level. The analysis of patient demographics has been proven useful to determine the specific needs of patients and physicians in terms of use of healthcare resources, in order to develop measures that allow reflexing disease complexity [8]. To facilitate such analyses, healthcare providers collect detailed information on healthcare usage. In Spain, the Catalan agency for quality and sanitary evaluation (AQuAS) recently implemented a new program to register this data, the program of data analysis for research and innovation in health (PADRIS) [9]. Consequently, the aim of this study was to develop a retrospective multicentre analysis via the records registered in PADRIS to scrutinise SMA patients’ demographics and disease management in Catalonia between 2007 and 2017. A secondary objective of the study was to validate the utility of PADRIS records prior to the program’s full operation.

## Methods

The study analysed records of all patients with a diagnosis of SMA in the region of Catalonia (7.5 million inhabitants) between 2007 and 2017. Data was obtained from project PADRIS, managed by AQuAS, via ethics committee approval. The database includes detailed information of healthcare usage, comprising primary care centres, hospitals (inpatient and outpatient care), extended care facilities and mental health centres. Records in the database are validated automatically via the evaluation of data consistency. Patient diagnoses and procedures were determined by means of the 9th revision of the International Statistical Classification of Diseases and Related Health Problems (ICD-9). When necessary, the extraction of single-patient information was carried out by eliminating repeated records corresponding to separated admissions, relying on the first admission as the index event.

The classification assigns patients an adjusted morbidity group (GMA), according to the number of systems they have affected by chronic diseases, and into 5 levels of complexity or risk. As it increases, GMA level correlates with a higher number of healthcare admissions of these patients and major pharmaceutical costs [10].

Data presentation is mainly descriptive. All statistical analyses were performed using Microsoft Excel© Professional Plus 2010 (Microsoft Corporation, Redmond, WA, USA).

## Results

### Patient characteristics

The ICD9 codes analysed represented SMA types I and II individually, and types II and IV together in the same cluster. Progressive SMA was considered independently, and a separate group designated the cases where SMA type was not specified (Table 1).

**Table 1:** SMA types claimed by ICD9 codes

Disease classification	ICD9 code
SMA-I. Werdnig-Hoffmann syndrome	335.0
SMA-III. Kugelberg-Welander syndrome	335.11
SMA-II and SMA-IV	335.19
Progressive SMA and Duchenne-Aran	335.21
Unspecified SMA	335.10

The first set of analyses examined patient records corresponding to 524 patients, most of them diagnosed with a progressive SMA. As for other SMA types, clear trends could not be detected due to the database characteristics. A clear sex bias was observed for most SMA types, with a proportion 60 to 40% male-female. SMA-II did not exhibit this pattern. Patients with no specified sex represented between 1 and 5% of the total (Table 2).

**Table 2:** Characteristics of the population of study

	Patient number	% of patients	% of male patients	% of female patients	Patients age ± SD
Total	524		58.02	40.84	38.31 ± 24.49
SMA-I	38	6.34	60.53	36.84	1.81 ± 3.01
SMA-III	84	14.02	51.19	45.24	25.11 ± 25.87
SMA-II and SMA-IV	62	10.35	61.29	33.87	58.25 ± 20.63
Progressive SMA	229	38.23	56.77	38.86	58.32 ± 23.61
Unspecified SMA	111	18.53	59.46	38.74	48.04 ± 27.00

Patients’ first admission with a SMA diagnosis was examined to estimate average age of diagnosis. The test revealed an average age of diagnosis of 38.31 ± 24.49 years. Differences between males and females were not evident for this parameter.

The database made available data on patients deceased during the study period, which allowed a rough estimation of mortality and an analysis of patients age at time of death (Table 3). Likewise, a qualitative analysis of the time elapsed between diagnosis and decease was considered relevant. Patients with SMA-I died 2 months to 3 years after diagnosis and patients with SMA-III died on average 6 months after diagnosis. Longer times elapsed for SMA-II, SMA-IV and progressive SMA, averaging between 4 and 5 years. An average of 22 patients died per year during the study period.

**Table 3:** Patients deceased during the studied period

	Patient number	% of patients	% of male patients	% of female patients	Patients age ± SD
Total	226		50.00	32.24	62.96 ± 25.41
SMA-I	17	44.74	47.83	42.86	0.50 ± 0.91
SMA-III	15	17.86	71.31	10.53	63.91 ± 24.33
SMA-II and SMA-IV	26	41.94	44.74	38.10	71.73 ± 13.26
Progressive SMA	110	48.03	53.08	40.45	67.25 ± 20.69
Unspecified SMA	58	52.25	68.18	34.88	64.40 ± 22.76

**Fig. 1:**
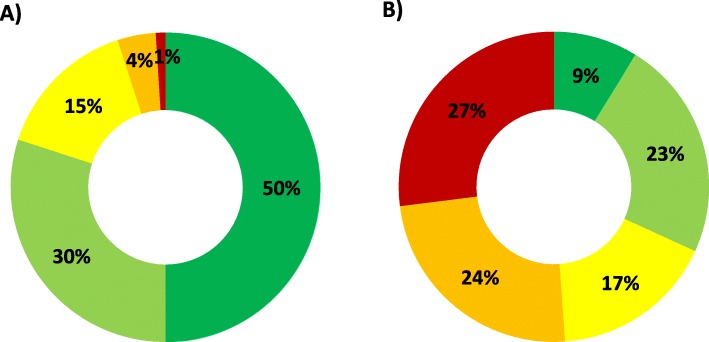
GMA health complexity levels. Risk 1 -green- low risk to 5 -red- very high risk in A) general population [10] and B) patients with SMA in 2016

The program assigns patients a GMA, according to the number of systems they have affected by chronic diseases, and are classified into 5 levels of complexity. The year 2016 (last available data) the population in this study was comprised by 71.26% of patients with 4 or more systems affected by a chronic condition, 17.53% of patients with 2 or 3 systems affected by a chronic condition, 6.32% of patients with an active neoplasm and 4.89% patients with 1 system affected by a chronic condition. As part of GMA calculation, patients were classified into 5 complexity or risk levels based of the general population as characterised in Fig. 1. The majority of patients with a SMA diagnosis would be represented in the very high risk level (27%) via the GMA population-based health risk stratification tool.

### Disease management

The classification of patient records permitted the analysis of separate episodes or independent admissions. The evaluation of disease management was based on a calculation of the services that attended the patients, the motivation of such admissions, an analysis of medical procedures and patient’s destination after discharge.

Most admissions were registered in primary healthcare centres, 36,724. Significantly smaller was the number of hospitalisations and outpatient consultations, 2518. A similar number of admissions corresponded to emergency room (ER) visits, 2836. Extended care facilities registered 659 admissions, and 13 records were obtained from mental care consultations. In the majority of admissions origin was indicated. 49.70% were scheduled admissions and 49.60% were urgent, the rest were unspecified. Considering all SMA-related admissions, the total number of admissions per patient was slightly superior in younger individuals. In patients aged 0 to 4 years, 3.0 admissions per patient were registered; in those aged 5 to 9 years 2.1 admissions were registered; in children aged 10 to 14 this parameter measured 1.7; finally, in patients older than 20 years of age it remained stable around the 1.2 admissions per patient.

Hospitalisation time averaged 8 days, excluding the stays in extended care facilities. As for patients’ destination after discharge, it was generally their residence, followed by transfers to extended care facilities.

On admission, patients were classified and diagnosed with a variety of symptoms and conditions, commonly with a direct link to SMA. Such conditions, excluding SMA, are listed in Table 4. Hospital admissions due to respiratory issues were significant in all groups; certain conditions as asthma or scoliosis were important hospitalisation motives in patients with infantile onsets of SMA. SMA-II and IV and progressive SMA, patients were often treated for diabetes mellitus type II and hypertension; equally, pressure ulcers appeared mostly in patients with progressive SMA. In order to determine the medical procedures chosen for these patients, admissions directly linked to SMA were selected. To this aim, the analysis included admissions with SMA diagnoses, those due to respiratory symptoms and other manifestations as dysphagia, anaemia, nutritional deficiencies and gastrointestinal issues.

**Table 4:** Number of admissions for other causes per SMA type

Diagnoses	SMA-I	SMA-III	SMA-II and IV	Progressive SMA
Abdominal pain	6	29	49	80
Acute bronchitis	43	70	19	2
Acute respiratory failure	29	15	7	31
Anxiety	11	25	27	120
Asthma, unspecified	106	262	76	54
Breath abnormality	39	74	4	37
Chronic bronchitis	39	45	33	198
Chronic respiratory failure	8	13	2	31
Diabetes mellitus type II	37	254	526	2295
Disuse muscular atrophy	33	79	20	46
Infantile cerebral palsy, unspecified	65	68	0	32
Lumbar pain	1	8	12	58
Malaise and fatigue	1	2	14	57
Obesity	2	52	11	112
Pulmonary collapse	27	41	1	3
Pressure ulcer	10	15	96	258
Quadriplegia	7	8	25	26
Scoliosis	19	49	5	19
Supplementary oxygen dependency	7	17	0	10
Unspecified essential hypertension	83	284	590	1368
Patient number	38	84	62	229

The aforementioned admissions took place mostly into hospitals (inpatient and outpatient care) and ER, and the most representative procedures were associated to respiratory difficulties (Table 5). Nursing care procedures were predominant in primary healthcare centres, focusing on sanitary education regarding the disease, the prescribed medication, diet or exercise, and the administration of intramuscular medication.

**Table 5:** Most common procedures in SMA-related admissions

Procedure	Hospital admissions	ER admissions	Total admissions
Antibiotic injection	3165	11	3888
Oxygen enrichment	2328	7	3004
Routine thorax radiography	1935	308	2619
Mechanical ventilation	1963	9	2581
Respiratory medication	1654	88	2231
Electrolyte injection	1388	25	1858
Electrocardiography	1311	150	1640
Arterial blood gas measurement	1105	41	1375
Steroid injection	1018	17	1293
Respiratory exercise	727	0	983
Blood microscopical analysis	298	483	908
Feeding tube	707	0	881
Transesophageal echocardiography	737	0	850
Tracheal intubation	461	0	548
Reconstructive surgery; pelvic bones	316	0	461
Internal fixation; pelvic bones	266	0	382

## Discussion

Interest in disease management data and patient characteristics has drastically increased in the past years. The analysis of patient records has been effective to orientate healthcare providers and governments and allow the application of measures that translate disease complexity [11]. Herein, the PADRIS program allowed the assessment of SMA current status in the region of Catalonia in consideration of patient demographics and disease management. The 524 patients included in the study were diagnosed with SMA between the years 2007 and 2017. Findings regarding patients’ sex distribution are consistent with those in prior studies. SMA is generally presented in higher frequencies in males, often near the 60% [12]. Similar observations were made in previous studies, in which patients’ sex seemed to play a role in disease severity [13, 14], although to this moment the mechanism of modulation has not been fully understood. A previous study in Spain suggested that it was the acute form of this disorder (SMA-I) the only variant affecting mostly females [15]. Most of the patients in the study were diagnosed with a progressive SMA, explainable by the lower specificity of its ICD9 classification. Given the characteristics of the database, data from this study cannot be used to infer disease prevalence, which poses as one of the limitations of database retrospective analyses.

Patients’ average age of diagnosis displayed reasonable variations among SMA types. SMA-I was diagnosed on average at 1.81 years, which is slightly elevated [5]. Likewise, average age of diagnosis of SMA-III, with a typical juvenile onset, is 25.11 years. Inevitably, delays due to miscoding cannot be ruled out. Still, the relevance of paediatric onsets appears tangible in the number of admissions registered per patient, superior in younger patients. This is not surprising given the gravity of SMA-0 and SMA-1, the variants with the earliest onsets [5]. Equally, age of decease generally correlates with previous findings. Patients with SMA-I typically die within the first 2 years of life [5], while patients affected with SMA-II, SMA-III and SMA-IV tend to die during their adult life. As for the time elapsed between diagnosis and decease, a relative small percentage of patients was available for consideration, allowing misconceptions. However, it is interesting to remark the average survival of patients with a progressive SMA, which correlates with the 4.6 years median survival displayed in a previous disease course study [2].

Novel information was provided by means of the GMA health risk stratification system. A rough assessment of patients’ relative risk provides a tool to refine disease evaluations and predict results in the use of health resources [10]. Analyses prove that as many as 71.26% of patients with SMA are affected by 4 or more chronic diseases in distinct organs, a percentage that measured 14.9% in the general population, which is likely to translate into a more intensive use of healthcare resources [10]. The study of multimorbidity groups will be crucial to evaluate patients’ healthcare needs in an effort to promote preventive care, taking into consideration that direct medical costs can account for at least 30% of all costs associated with a patient with SMA [16, 17].

In this context, and in terms of disease management, current available information reflects the predominant use of SMA patients of primary healthcare, although most diagnosis and treatment procedures are performed in specialised healthcare facilities, both in and outpatient care. The majority of these admissions were related to respiratory issues derived from the disease, in addition to any patients with a diagnosis of SMA with respiratory distress that could contribute to this outcome [18]. Additional chronic conditions as type II diabetes or essential hypertension registered on admission confirm the weight of multimorbidity in patients’ use of resources.

Nonetheless, procedures aimed to resolve respiratory problems made up for the majority of measures. Oxygen enrichment and mechanical ventilation are crucial when patients present respiratory muscle weakness and still are the preferred treatment in such cases [19, 20]. Other repeated procedures are designed to reduce motor impairment, as the fixation or reconstruction of pelvic bones, an expected outcome given the recommendation for intervention in these patients to diminish the consequences of scoliosis, surgery that appears in the highest percentages in the youngest patients to lose ambulation capacity [4, 21].

It is plausible that a number of limitations may have influence in the results obtained. Data recording in the distinct healthcare sources started at varied time points between 2007 and 2012, and the restricted use of accurate ICD9 definitions during the first studied years left a considerable amount of data unspecified. Moreover, the use of ICD9 for codification in the database limits the relevance of the results; the lack of specific codes for SMA-II and SMA-IV impede further result interpretation. Measures were taken to avoid errors derived from miscoding; nonetheless the complete avoidance of data replication cannot be ensured. Presumably, data herein and further analysis of the database will provide new information regarding SMA management in the past years, facilitating the establishment of improved health protocols, moving towards P4 Medicine: Personalized, Predictive, Preventive and Participatory.

## Conclusions

This study uses the newly established database PADRIS to assess the current status of SMA in the region of Catalonia. The 524 patients with a diagnose of SMA exhibited few differences with patients in other European countries. Patients were treated for SMA mainly in hospitals, and the use of healthcare resources was focused on resolving respiratory issues, and to a lesser extent, to diminishing the consequences of scoliosis. Patients presented a higher risk than the general population associated with an elevated number of admissions in health centres and ER, in addition to the elevated incidence of multimorbidities.

## Data Availability

Data not included in this submission due to legal stipulations from the Catalan Health Department.

## Abbreviations

AQuAS: Catalan agency for quality and sanitary evaluation (Agència de Qualitat i Avaluació Sanitàries de Catalunya)
ER: Emergency Room
GMA: Adjusted Morbidity Groups
ICD9: 9th revision of the International Statistical Classification of Diseases and Related Health Problems
PADRIS: Program of data analysis for research and innovation in health (Programa d’Analítica de Dades per a la Recerca i la Innovació en Salut)
SMA: Spinal Muscular Atrophy
